# Mental health service use among adolescents in three low- and middle-income countries: An analysis of the National Adolescent Mental Health Surveys

**DOI:** 10.1186/s13034-025-00924-2

**Published:** 2025-07-31

**Authors:** Amirah Ellyza Wahdi, Yufan Putri Astrini, Althaf Setyawan, Shoshanna L. Fine, Astha Ramaiya, Mengmeng Li, Yohannes D. Wado, Vu Manh Loi, Joemer C. Maravilla, James G. Scott, Siswanto Agus Wilopo, Holly E. Erskine

**Affiliations:** 1 Center for Reproductive Health, Faculty of Medicine, Public Health, and Nursing, Universitas Gadjah Mada, Yogyakarta, Indonesia; 2 Department of Biostatistics, Epidemiology, and Population Health, Faculty of Medicine, Public Health, and Nursing, Universitas Gadjah Mada, Yogyakarta, Indonesia; 3 Department of Mental Health, Johns Hopkins Bloomberg School of Public Health, Johns Hopkins University, Baltimore, MD USA; 4 Department of Population, Family and Reproductive Health, Johns Hopkins Bloomberg School of Public Health, Johns Hopkins University, Baltimore, MD USA; 5 African Population and Health Research Center, Nairobi, Kenya; 6 Institute of Sociology, Vietnam Academy of Social Sciences, Hanoi, Vietnam; 7 School of Public Health, The University of Queensland, Herston, QLD Australia; 8 Queensland Centre for Mental Health Research, Wacol, QLD Australia; 9 Institute of Health Sciences and Nursing, Far Eastern University, Manila, Philippines; 10 Child Health Research Centre, The University of Queensland, South Brisbane, QLD Australia; 11 Child and Youth Mental Health Service, Children’s Health Queensland, South Brisbane, QLD Australia; 12 Institute for Health Metrics and Evaluation, University of Washington, Seattle, WA USA

**Keywords:** Adolescent, Mental health, Service use, Kenya, Indonesia, Vietnam, Low- and middle-income countries

## Abstract

**Background:**

Mental disorders are prevalent and their onset is highest during adolescence. However, there are limited data on adolescent mental health service utilization in low- and middle-income countries.

**Methods:**

Data were from the National Adolescent Mental Health Surveys (NAMHS), nationally representative household surveys of adolescents aged 10–17 years and their primary caregiver conducted in Kenya, Indonesia, and Vietnam. All primary caregivers were asked whether their adolescent used any services providing support or counselling for emotional or behavioural problems in the past 12 months. Mental disorders were assessed using the Diagnostic Interview Schedule for Children, Version 5 (DISC-5). The prevalence of service use was calculated among those with mental disorders, subthreshold mental disorders, and no mental disorder. The prevalence of service use among those with either a diagnostic or subthreshold mental disorder was compared by demographic characteristics and broad mental disorder type. All estimates were weighted using population weights for each country and presented with 95% confidence intervals (CI).

**Results:**

Very few adolescents with a mental disorder (Kenya: 11.9%, 95% CI: 9.3–15.1; Indonesia: 4.7%, 95% CI: 1.9–11.1; Vietnam: 8.2%; 95% CI: 3.9–16.4) or a subthreshold mental disorder (Kenya: 10.8%, 95% CI: 9.1–12.9; Indonesia: 2.2%, 95% CI: 1.1–4.5; Vietnam: 8.5%; 95% CI: 5.0–14.1) accessed services that provide support or counselling for emotional or behavioural problems in the past 12 months. In Kenya, being older (aOR 1.41, 95% CI; 1.07–1.86) and female (aOR 1.77, 95% CI; 1.34–2.34) were associated with increased odds of service use, while having internalising disorders only (aOR 0.45, 95% CI; 0.30–0.65) was associated with decreased odds of service use. No difference by demographic characteristics or mental disorder type was seen in Indonesia and Vietnam.

**Conclusions:**

Only a small proportion of adolescents with a diagnostic or subthreshold mental disorder accessed services for mental health in Kenya, Indonesia, and Vietnam. These findings indicate the need for greater support for adolescents with mental disorders and provide critical context for governments and relevant in-country stakeholders when reviewing the availability and accessibility of adolescent mental health services.

**Supplementary Information:**

The online version contains supplementary material available at 10.1186/s13034-025-00924-2.

## Background

Adolescence is a crucial development period, marked by profound biological, cognitive, psychosocial, and emotional changes that shape a young person’s trajectory into adulthood [1, 2]. In parallel, mental disorders, and poor mental health more broadly, can significantly impact both an adolescent’s transition into adulthood and their health and well-being later in life [3]. Mental disorders tend to have their highest incidence during adolescence [4, 5] and a recent systematic review estimated the global pooled prevalence of mental disorders at 13.4% among children and adolescents [6].

While nationally representative studies reporting adolescent mental health service use are available, most are from high-income countries (HICs) [7]. Evidence from the United States shows that service use among adolescents with mental disorders is relatively low. For example, the 2019 National Survey of Drug Use and Health (NSDUH) found that of the 15.8% of adolescents aged 12–17 years who had a major depressive episode in the past year, less than half (45.8%) had accessed mental health treatment [8]. An analysis of the 2011–2018 NSDUH showed similar results, with only 49% of those adolescents reporting a major depressive episode in the past year accessing some care for their symptoms during the same period [9]. Previous national studies in the US found even greater gaps in service use when factoring in other mental disorders. For example, the 2001–2004 National Health and Nutrition Examination Survey (NHANES) found that only 50.6% of 8-15-year-olds with mental disorders accessed care from a hospital, clinic, or office because of their disorder in the past 12 months [10]. The 2002–2004 National Comorbidity Survey-Adolescent Supplement (NCS-A) found that among 13-18-year-olds with mental disorders, only 36.2% had received any mental health service, with this proportion increasing only somewhat for those with severe disorders (47.4%) [11].

These findings from the US are in line with studies from other HICs, such as the 2014 Young Minds Matter study in Australia, which found that only 44.7% of 13-17-year-olds meeting diagnostic criteria for a mental disorder in the past 12 months reported accessing services for emotional or behavioural problems during the same period [12]. Another population-based longitudinal study involving 12,512 adolescents aged 13–14 years old from Sweden showed that 16.1% of girls and 15.8% of boys with moderate mental health symptoms used mental health services [13]. The percentage increased to 28.6% and 23.6% among girls and boys with severe symptoms, respectively [13].

Compared to HICs, few population-based surveys reporting adolescent mental health service use are available for low- and middle-income countries (LMICs) [14], despite the majority of the globe’s adolescents (73%) residing in these regions [15]. Most studies reporting mental health service use in LMICs tend to be limited to adults [16, 17] or focused on specific adolescent subpopulations, such as adolescents living with human immunodeficiency virus (HIV) and adolescents in resource-constrained settings [14, 18]. Of the limited population studies available for adolescents, most, if not all, point towards very low rates of mental health service use. An analysis from the Brazilian High-Risk Cohort (BHRC), a study of 1,400 adolescents in Sao Paulo and Porto Alegre, found that only 22.4% of those with a mental disorder used a service in the past twelve months, with this proportion slightly increasing to 27.5% among adolescents with a persistent disorder [19]. Similarly, a representative study of adolescents living in Mexico City, Mexico, reported that only 13.7% of 12-17-year-olds with mental disorders received mental health services in the past 12 months [20]. Other available mental health service use data tends to come from treated populations, i.e., those who have generally accessed services from mental health outpatient facilities, rather than data for the whole population which can then give an indication of unmet need. For example, an analysis of pooled data from 42 LMICs found a median one-year treated prevalence for children and adolescents of 159 per 100,000, compared to 664 per 100,000 for adults, with the authors further reporting that only 3% of mental health outpatient facilities were reserved for children and adolescents specifically [21].

Given that the onset of mental disorders is highest during adolescence [4, 5], the low proportion of adolescents accessing mental health services highlights a potential missed opportunity for early intervention [22]. Data on mental health service utilisation by adolescents, in conjunction with equivalent data reporting on the prevalence of mental disorders among the general adolescent population, is vital for policymakers and service planners to make informed decisions about service provision as well as to identify potential barriers to care and gaps in availability. However, without representative data for mental health service use among adolescents in LMICs, it is nearly impossible to understand the true unmet need for mental health support among adolescents. Standardized data on mental health service use are critical for effectively allocating resources, particularly in LMICs where health resources are limited and multiple competing priorities remain, including communicable diseases [23]. Data specific to LMICs is essential to inform in-country decision-making, given that the individuals or sectors providing mental health support may differ from HICs, e.g., where faith or spiritual leaders may be a primary source of mental health support, while different cultural factors may impact an adolescent’s desire or ability to seek services, e.g., in terms of stigma [24] and resources availability [25]. Further, representative data allows cross-country comparison and monitoring of progress toward global development goals in adolescent mental health [26, 27].

The National Adolescent Mental Health Surveys (NAMHS) were specifically designed to address the gaps in knowledge about adolescent mental health in LMICs. NAMHS involved nationally representative household surveys of mental disorders among adolescents aged 10–17 years in three LMICs: Kenya, Indonesia, and Vietnam. The 12-month prevalence of mental disorders among adolescents reported by NAMHS was 12.1%, 5.5%, and 3.3% in Kenya, Indonesia, and Vietnam, respectively [28]. In addition to the diagnostic measure of mental disorders, the NAMHS instrument included questions about the adolescent’s use of services for emotional or behavioural problems. As such, NAMHS provides the opportunity to assess service use and unmet need in national surveys conducted in parallel in three LMICs. This study examines mental health service use among adolescents in Kenya, Indonesia, and Vietnam, including among those with a diagnostic mental disorder or a subthreshold mental disorder, with this further disaggregated by key demographic characteristics and broad mental disorder type.

## Methods

### Data source

This study utilised data from NAMHS which provides nationally representative data of adolescent mental disorders from household surveys of adolescents and their primary caregiver in Kenya, Indonesia, and Vietnam [28, 29]. Sampling frames able to generate nationally representative samples were designed by each in-country NAMHS team in collaboration with relevant in-country statistical agencies, further utilising available census data and data from large-scale surveys [28]. A stratified multistage clustered random sample design utilising hierarchical administrative and/or geographical levels relevant to the specific country was employed. Households with adolescents aged 10–17 years were randomly selected to participate and one adolescent was randomly chosen to participate within households with more than one eligible adolescent. Eligible adolescents were those who lived at the household most of the time, lived with their primary caregiver, were not married, and spoke the language of the survey. Further, a brief measure of disability was administered to ensure adolescents did not have a physical or cognitive condition that would stop them from meaningfully participating in the survey. Full sampling information and eligibility criteria have been published elsewhere [28, 29]. The survey took place in 2021 and was conducted by trained lay interviewers using a smartphone or tablet. Median interview times ranged from 35 to 45 min for primary caregivers and 49 to 64 min for adolescents [28]. Consent from the primary caregiver and assent from the adolescent was obtained prior to the interview, and privacy was a requirement of participation. In total, 5,155, 5,664, and 5,996 households completed the interviews in Kenya, Indonesia, and Vietnam, respectively. The response rates were 97.7% for Kenya, 92.2% for Indonesia, and 81.1% for Vietnam. The detailed methodology of NAMHS has been described elsewhere [28, 29].

### Measures

Detailed information on the NAMHS instrument, including its comprehensive cultural adaptation, has been previously published [28, 29]. Briefly, measures were either adapted from existing measures or developed specifically for NAMHS. Measures were translated into the respective language of the country and underwent back-translation to English, were reviewed by in-country clinicians, underwent adjustment after the pilot study phase, and were subject to ongoing revisions over an intensive 18-month period prior to data collection. The revisions included specific consideration of the culture of each country, e.g., ensuring behaviours/experiences/situations described in the questions were relevant to that country, while maintaining conceptual consistency to allow for cross-national comparison. The following sections describe the measures relevant to this study.

#### Service use

NAMHS defined services as any support or counselling the adolescent received for emotional or behavioural problems in the past 12 months, with no specific requirement that these services exist within the health or education setting. The scope of what was considered a ‘mental health service’ was intentionally broad to capture the full scope of services relevant to mental health, particularly within LMICs, e.g., services received from religious or faith leaders.

Mental health service use by adolescents was assessed by asking the primary caregiver the following question: *“In the past 12 months*,* has [your adolescent] used any services that provide support or counselling for emotional or behavioural problems? This includes services received at school*,* at a community clinic*,* from a doctor*,* or a religious/faith leader.”* This question was asked to all primary caregivers regardless of the adolescent’s mental disorder status.

#### Mental disorders

Adolescent mental disorders were measured using the Diagnostic Interview Schedule for Children, Version 5 (DISC-5) [30], which assesses mental disorders according to the diagnostic criteria presented in the Diagnostic and Statistical Manual of Mental Disorders, Fifth Edition (DSM-5) [31]. The DISC-5 is designed to be administered by interviewers with no formal medical training but trained to administer the instrument. NAMHS assessed the presence of six mental disorders in the past 12 months: social phobia, generalized anxiety disorder (GAD), major depressive disorder (MDD), conduct disorder, post-traumatic stress disorder (PTSD), and attention-deficit/hyperactivity disorder (ADHD). These disorders were chosen due to their prevalence in adolescence [32], their inclusion in the Global Burden of Disease Study [32, 33], and input from the in-country NAMHS teams [29]. All diagnostic modules were administered to the adolescents except the ADHD module, which was administered to the primary caregiver.

Adolescent mental disorder status was categorised into three mutually exclusive groups following the DISC-5 scoring algorithms: diagnostic mental disorder (i.e., met DSM-5 diagnostic criteria for any of the six mental disorders in the past 12 months), subthreshold mental disorder (i.e., endorsing at least half of the symptoms required for diagnosis but not meeting DSM-5 diagnostic criteria for any mental disorder in the past 12 months), and no mental disorder (i.e., not meeting requirements for either a diagnostic mental disorder or a subthreshold mental disorder in the past 12 months).

#### Demographics

All demographic information was reported by the primary caregiver, including adolescent age (grouped as 10–14 years and 15–17 years), adolescent sex (i.e. male or female), and information relating to household wealth as measured by the wealth index. Household wealth was converted into wealth quintiles which were generated by weighting household assets according to a standardized methodology developed for NAMHS [28]. Urbanicity (i.e., urban and rural) was pre-determined by the household’s location which was incorporated into the sampling frame in each country [28].

Detailed demographic information for adolescents and primary caregivers has been published previously [28]. In each country, samples contained a relatively even proportion of male and female adolescents (weighted proportion of males: Kenya = 50.1%; Indonesia = 50.9%; Vietnam = 52.0%). The average age of adolescents in the sample was 13 years in all three countries. In Kenya and Vietnam, approximately two thirds of households were located in rural areas while the inverse was true of Indonesia. Comparison to recent census data in each country demonstrated that the demographics of the samples reflected the national population [28].

### Data analysis

Adolescent mental health service use was calculated as a proportion of the whole sample and then further disaggregated by mental disorder status in the past 12 months. All non-meaningful responses (‘Don’t know’ or ‘Prefer not to say’) were omitted from the analysis, with the proportion of non-meaningful responses as follows in each country: Kenya: 0.1%; Indonesia: 0.8%; Vietnam: 2.0%. Multivariable logistic regression was applied to estimate adjusted odds ratios (aORs) for mental health service use among adolescents (i.e., yes or no) with any diagnostic or subthreshold mental disorder in the past 12 months by demographic characteristics (i.e., age group, sex, urbanicity, and household wealth) and broad mental disorder type (grouped as internalising disorders only, externalising disorders only, and both internalising and externalising disorders). Internalising disorders included social phobia, GAD, MDD, and PTSD while externalising disorders included ADHD and conduct disorder. All estimates were weighted using country-specific population weights, whereby inverse probability weighting was applied to generate nationally representative estimates with corresponding 95% confidence intervals [28]. All analyses were conducted using Stata17 [34].

## Results

Table 1 shows the proportion of adolescents who used any service providing support or counselling for emotional or behavioural problems in the past 12 months in Kenya, Indonesia, and Vietnam (corresponding unweighted numbers are available in Table S1 in Additional File 1). Service use was low in all three countries, although statistically significant differences were seen, with Kenya having the highest proportion of service use (8.7%, 95% CI: 7.6–10.0), followed by Vietnam (6.6%, 95% CI: 4.5–9.6) and Indonesia (2.0%, 95% CI: 1.1–3.7). Within country, only Kenya showed a statistically significant difference in service utilization between adolescents with any diagnostic mental disorder (11.9%, 95% CI: 9.3–15.1) or any subthreshold mental disorder (10.8%, 95% CI: 9.1–12.9) as compared to adolescents with no mental disorder (6.8%, 95% CI: 5.7–8.2). While a similar pattern was seen in Indonesia and Vietnam, these differences were not statistically significant.

**Table 1:** Proportion of adolescents who used any service providing support or counselling for emotional or behavioural problems in the past 12 months by mental disorder status in Kenya, Indonesia, and Vietnam

Mental disorder status	Kenya, % (95% CI)	Indonesia, % (95% CI)	Vietnam, % (95% CI)
Overall*	8.7 (7.6–10.0)	2.0 (1.1–3.7)	6.6 (4.5–9.6)
Any mental disorder	11.9 (9.3–15.1)	4.7 (1.9–11.1)	8.2 (3.9–16.4)
Any subthreshold mental disorder	10.8 (9.1 12.9)	2.2 (1.1–4.5)	8.5 (5.0–14.1)
No mental disorder	6.8 (5.7–8.2)	1.6 (0.8–3.2)	6.1 (4.1–9.1)

*Total sample after omitting non-meaningful responses (i.e., Don’t know or Prefer not to say) to the question regarding use of any service providing support or counselling for emotional and behavioural problems in the past 12 months

Mental health service use among adolescents with any diagnostic mental disorder in the past 12 months did not significantly differ across countries (Kenya:11.9%, 95% CI: 9.3–15.1; Indonesia: 4.7%, 95% CI: 1.9–11.1; Vietnam: 8.2%, 95% CI: 3.9–16.4). Among adolescents with any subthreshold mental disorder and with no mental disorder, only Indonesia (2.2%, 95% CI: 1.1–4.5 and 1.6%, 95% CI: 0.8–3.2, respectively) demonstrated significantly lower proportions of adolescents using services compared to Kenya (10.8%, 95% CI: 9.1–12.9 and 6.8%, 95% CI: 5.7–8.2, respectively) and Vietnam (8.5%, 95% CI: 5.0–14.1 and 6.1%, 95% CI: 4.1–9.1, respectively).

Table 2 shows the combined proportion of adolescents with any diagnostic or subthreshold mental disorder who accessed services for emotional or behavioural problems in the past 12 months disaggregated by demographic characteristics as well as broad mental disorder type (the corresponding unweighted numbers are available in Table S2 in Additional File 1). Only Kenya demonstrated significant differences in service use by any demographic characteristics, specifically only by adolescent sex, with females significantly more likely than males to have accessed services (13.7%, 95% CI: 11.2–16.7 vs. 8.7%, 95% CI: 7.0–10.7). In Kenya, adolescents with internalising disorders only (7.6%, 95% CI: 6.0–9.6) demonstrated significantly lower rates of service use compared to those with externalising disorders only (14.5%, 95% CI: 11.4–18.3) and those with both types of disorders (13.8%, 95% CI: 10.8–17.4). No significant differences were found by demographic characteristics or broad mental disorder type in Indonesia or Vietnam.

**Table 2:** Service use by adolescents with any diagnostic or subthreshold mental disorder in the past 12 months by demographic characteristics and broad mental disorder type in Kenya, Indonesia, and Vietnam

Characteristic	Kenya, % (95% CI)	Indonesia, % (95% CI)	Vietnam, % (95% CI)
Total	11.1 (9.4–13.1)	2.6 (1.4–4.9)	8.5 (5.4–13.2)
Age			
10–14 years	10.2 (8.4–12.4)	2.0 (1.0–4.0)	8.1 (4.9–13.1)
15–17 years	13.0 (10.5–15.9)	3.9 (1.9–7.8)	9.1 (5.2–15.5)
Sex			
Male	8.7 (7.0–10.7)	3.2 (1.7–5.9)	10.4 (5.9–17.6)
Female	13.7 (11.2–16.7)	2.1 (1.0–4.3)	6.6 (3.9–11.0)
Urbanicity			
Rural	13.7 (10.9–17.1)	2.7 (1.2–6.0)	6.8 (3.8–11.8)
Urban	9.2 (7.3–11.6)	2.5 (1.0–6.3)	9.1 (5.1–15.7)
Wealth quintile			
1– lowest	7.1 (4.1–11.9)	1.8 (0.7–4.4)	9.2 (2.8–26.2)
2	9.2 (6.1–13.6)	2.3 (1.0–5.3)	6.5 (3.4–11.9)
3	10.5 (7.8–14.0)	1.9 (0.6–6.2)	7.2 (3.5–14.2)
4	15.4 (11.7–20.0)	4.5 (1.5–12.7)	8.7 (3.6–19.5)
5– highest	11.6 (8.9–15.0)	3.4 (1.2–9.1)	12.5 (5.8–25.0)
Mental disorder type ^a^			
Externalising only	14.5 (11.4–18.3)	1.7 (0.6–4.7)	10.7 (5.1–21.2)
Internalising only	7.6 (6.0–9.6)	2.9 (1.4–6.1)	7.9 (4.5–13.4)
Both	13.8 (10.8–17.4)	2.6 (1.0–6.4)	13.0 (5.6–27.5)

^a^Mental disorder refers to both diagnostic mental disorders and subthreshold mental disorders in the past 12 months. Internalising mental disorders include social phobia, GAD, MDD, and PTSD. Externalising disorders include ADHD and conduct disorder. “Both” refers to adolescents with both types of mental disorder. Adolescents could have more than one mental disorder

As shown in Table 3, the results of the logistic regression were consistent with the reported differences in prevalence (Table 2) in Kenya seen by demographic characteristics and broad mental disorder type, with the exception of age group where older adolescents (aOR 1.41, 95% CI; 1.07–1.86) were significantly more likely to have used services in the past 12 months than younger adolescents. In Kenya, females (aOR 1.77, 95% CI; 1.34–2.34) were significantly more likely than males to have used services while adolescents with internalising disorders only (aOR 0.45, 95% CI: 0.30–0.65) having significantly lower odds of using services than adolescents with externalising disorders only. No significant difference in service use was seen by urbanicity or wealth quintile in Kenya. No significant differences were found for service use by any demographic characteristic or broad mental disorder type in Indonesia or Vietnam.

**Table 3:** Adjusted odds ratios for service use by adolescents with any diagnostic or subthreshold mental disorder in the past 12 months by demographic characteristics and broad mental disorder type in Kenya, Indonesia, and Vietnam

Characteristics	Kenya	Indonesia	Vietnam
	aOR (95% CI)	aOR (95% CI)	aOR (95% CI)
Age			
10–14 years (ref)	1	1	1
15–17 years	1.41 (1.07–1.86)	1.95 (0.93–4.09)	1.16 (0.70–1.93)
Sex			
Male (ref)	1	1	1
Female	1.77 (1.34–2.34)	0.62 (0.37–1.06)	0.64 (0.32–1.27)
Urbanicity			
Rural (ref)	1	1	1
Urban	0.79 (0.51–1.25)	1.17 (0.31–4.38)	1.59 (0.65–3.87)
Wealth quintile			
1 - lowest (ref)	1	1	1
2	1.24 (0.61–2.52)	1.29 (0.34–4.91)	0.76 (0.26–2.18)
3	1.36 (0.68–2.74)	1.14 (0.20–6.45)	0.88 (0.21–3.75)
4	1.91 (0.90–4.03)	2.71 (0.62–11.89)	1.21 (0.26–5.56)
5 - highest	1.36 (0.65–2.84)	2.03 (0.42–9.88)	1.72 (0.37–8.03)
Mental disorder type ^b^			
Externalising only (ref)	1	1	1
Internalising only (ref)	0.45 (0.30–0.65)	1.72 (0.53–5.58)	0.76 (0.24–2.39)
Both	0.90 (0.63–1.30)	1.79 (0.54–5.89)	1.21 (0.34–4.35)

^a^ Each odds ratio was adjusted for all other characteristics i.e., age, sex, urbanicity, wealth quintile, and broad mental disorder type

^b^ Mental disorders refers to both diagnostic mental disorders and subthreshold mental disorders. Internalising mental disorders include social phobia, GAD, MDD, and PTSD. Externalising disorders include ADHD and conduct disorder. “Both” refers to adolescents with both types of mental disorder. Adolescents could have more than one mental disorder

## Discussion

The current study, based on nationally representative data across three countries, found that service use was low across Kenya, Indonesia, and Vietnam. Very few adolescents with either a diagnostic or subthreshold mental disorder had accessed services that provide support or counselling for emotional or behavioural problems in the past 12 months. Among those adolescents with a mental disorder, less than 1 in 8 used services in any country, ranging from 4.7% in Indonesia to 11.9% in Kenya. The equivalent proportion among those with a subthreshold mental disorder showed the same pattern, ranging from 2.2% in Indonesia to 10.8% in Kenya. Among those with a mental disorder or those who were subthreshold for a mental disorder, only Kenya demonstrated differences in service use by demographic characteristics when controlling for all others, with older adolescents (aOR: 1.40) and females (aOR: 1.77) more likely to have accessed services than young adolescents and males, respectively. Service use among adolescents in Kenya also differed by broad mental disorder type, with those with internalising disorders only (aOR: 0.45) being significantly less likely to have accessed services than those with externalising disorders only and with externalising and internalising disorders.

The findings of NAMHS point towards a substantial unmet need for mental health services in Kenya, Indonesia, and Vietnam, with over 85% of adolescents with either a diagnostic mental or subthreshold mental disorder not accessing services in the past 12 months. This is much greater than the already high unmet need reported in previous studies of HICs, such as the 2019 NSDUH, where only 45.8% of adolescents with a major depressive episode had accessed mental health care in the past year [8]. Studies of mental health service use from LMICs are extremely limited, even more so for adolescents, although those that are available also indicate a substantial unmet need in adolescents. A study in a low-income urban area in Brazil found that only 12% of young people aged 6–15 years with a mental health problem had accessed mental health services in the past year [35]. The differences in service use reported by NAMHS as compared to other, albeit limited, studies from LMICs, highlights the importance of using country-specific data to inform national policy rather than relying more broadly on data from surrounding regions or HICs.

The Brazilian study of a low-income urban area also found that young males were significantly more likely than females to have accessed services (aOR: 2.9) [35]. This contrasts with NAMHS, where females in Kenya were more likely to have accessed services (aOR: 1.7), while no significant difference in service use by sex was seen in Indonesia or Vietnam. Existing studies from HICs support the pattern reported for Kenya in NAMHS, i.e., finding that older and female adolescents access mental health care services more often than younger and male adolescents [36]. However, studies from HICs have also reported higher service use among those with internalising disorders as compared to externalising disorders [37, 38], contrasting with the NAMHS findings in Kenya. However, given internalising disorders are generally more prevalent among females than males [39], this finding indicates that other factors are potentially influencing an adolescent’s use of mental health services, from individual-level factors e.g., an adolescent’s relationship with their primary caregiver, through to more structural factors e.g., health system structure and payment model. The suite of measures included in NAMHS [28] provides an opportunity to investigate these factors in future analyses.

The operational definition of mental health services within NAMHS included support and counselling for emotional or behavioural problems provided outside the health sector, such as those offered by educators and faith/religious leaders. Even with this inclusion, the rates of mental health service use by adolescents in the past 12 months was low for all three countries. Interestingly, a small proportion of adolescents with no mental disorder in Kenya (6.8%), Indonesia (1.6%), and Vietnam (6.1%) still accessed support and counselling for emotional or behavioural problems. It is possible these adolescents or their caregivers were seeking support in a preventative manner or that the adolescent had emotional or behavioural problems that did not meet a subthreshold symptom level in the past 12 months.

While this analysis did not include an assessment of barriers to care, several possible explanations exist for the low rates of service use. Mental health literacy has been acknowledged as a critical aspect that influences mental health care utilization and is linked to perceived mental health care need [40, 41]. Numerous studies on mental health literacy in LMICs have shown that low mental health literacy is a key barrier to accessing professional mental health care [42]. A scoping review of child and adolescent mental health services in six African countries (Ethiopia, Mali, Egypt, South Africa, Nigeria, and Tunisia), where the adolescents reported they sought help from both professional and traditional/alternative services, found that a limited understanding of the biomedical and psychosocial causes of mental health problems among communities hindered mental health help-seeking [43]. Similar results were found in a study in Asia involving 1,002 adolescents [44] and, more specifically, in Indonesia [45] and Vietnam [46]. Another possible explanation for the low rates of service use reported in the current study is the pervasive stigma regarding mental health in LMICs. Fear of being treated differently, feeling ashamed of their adolescent’s condition, and attempting to hide their adolescent’s condition were some of the ways in which stigma was been reported as manifesting in previous studies [47]. Several studies in LMICs, including studies conducted in Kenya, Indonesia, and Vietnam, have identified stigma as a key barrier to accessing mental health care among children and adolescents [42, 43, 46, 48, 49]. A combination of low mental health literacy and mental health stigma among adolescents or their parents potentially contributed to the low rates of mental health service use observed in this study.

Furthermore, it is essential to note the lack of accessible health sector-based mental health care services available in Kenya, Indonesia, and Vietnam. Costs and lack of mental health care services will likely hinder mental health help-seeking [50], which could also explain the low prevalence of accessing mental health services. In Kenya, there were only approximately 116 psychiatrists, and less than 500 registered psychiatric nurses, concentrated in the capital city of Nairobi as of 2016 [51, 52]. The number of mental health providers specializing in children and adolescents is even smaller [53]. A similar situation was also found in Indonesia with only approximately 600 psychiatrists in the whole country and approximately three-quarters located in Java Island with 86% of these practicing in the capital city, Jakarta [54]. Similar information on the mental health workforce is not currently available for Vietnam. Among these three countries, only Indonesia has universal health coverage (UHC), which includes coverage for mental health problems [55]. In Kenya, the available premium-based health insurance is not affordable for most people [56]. In Vietnam, although the government has started a program to increase access to mental health care, it is intended to address more severe mental disorders, such as schizophrenia, and adolescents are not clearly stated as one of the beneficiaries [57]. The relative lack of adolescent mental health care available through the health sector highlights both the need and potential benefit of formally involving other sectors (e.g., education, religious, etc.) in adolescent mental health care.

There has been some promising progress at the policy level, although this differs by country. For example, the Kenya Mental Health Policy 2015–2030 [58] was developed in response to the need to reform mental health systems in Kenya and contains some broad policy directions regarding mental health service delivery e.g., strengthening the referral system. However, the policy only mentions adolescents in the context of establishing child and adolescent outpatient and inpatient facilities and does not contain a specific framework for adolescent mental health services at different levels of care. Nevertheless, such policy initiatives could be built upon or expanded to represent adolescent mental health specifically, further incorporating other efforts in Kenya to identify potential avenues for support and improvement [59]. In Indonesia, the Indonesian Mental Health Act [60] was passed in 2014, with the key objective to ensure the provision and improve the quality of mental health services [60]. Again, there is no specific policy pertaining to adolescent mental health nor the provision of corresponding services. Further, clinicians, stakeholders, and researchers have reported concerns regarding the Act’s implementation and coverage of key issues impacting mental health [61, 62], with these concerns potentially further exacerbated by the repeal and integration of the Act into the Omnibus Health Act of 2023 [63]. In Vietnam, Decision 1929/QD-TTg in 2020 approved community-based social assistance and rehabilitation programs for people with mental disorders [64]. However, adolescents are only mentioned in the context of subpopulations and the only focus on young people is in relation to children with autism. Similarly, the National Plan for the Prevention and Control of Non-Communicable Diseases and Mental Health Disorders for 2022–2025, issued by the Prime Minister in Decision 155/QD-TTg in 2022 [65], focuses only on schizophrenia and depression without attention to adolescent-specific strategies or other disorders prevalent during adolescence [66]. The findings of the current study provide impetus for building upon these existing policies and drafting new policies for adolescent mental health, with a focus on addressing the reported gap between mental disorder prevalence and service use.

While the findings from NAMHS represent an important and much needed contribution to better understand adolescent mental health service use, there are some key limitations that must be considered when interpreting the results. For example, the potential broad interpretation of the ‘emotional and behavioural problems’ as noted in the service use question is one such limitation of the current study. It is possible that ‘emotional or behavioural problems’ may have been interpreted as referring to a problem not reflective of a mental disorder or to a mental disorder not measured in NAMHS. However, given the corresponding low rates of service use among those with a mental disorder or subthreshold mental disorder, it does not appear that the inclusive nature of the question resulted in an overestimate of service use in any of the three countries. In parallel, questions have previously been raised about the applicability of Western-based diagnostic criteria for mental disorders within LMICs and whether these are too restrictive for determining the potential need for mental health support in the adolescent population [28]. Future analyses have the opportunity to investigate this further by adjusting scoring algorithms, given the comprehensiveness of the mental disorder measure, i.e., the DISC-5, utilized in NAMHS.

Other limitations also warrant consideration when interpreting the results. For example, the question regarding seeking services that provide support or counselling for emotional or behavioural problems was asked to the adolescent’s primary caregiver. Given parents and caregivers generally facilitate an adolescent’s service access, specifically in case of access to formal health services [67–69], it was determined that the primary caregiver would likely be the best respondent to this question. This decision was supported by a study in Indonesia involving 2,161 adolescents, which showed that the prominent reason adolescents forwent accessing professional services was their lack of knowledge of available services [70]. This study emphasized the necessity of others in facilitating adolescents’ access to professional services. However, it is possible that adolescents, particularly older adolescents, may have accessed services without their caregiver’s knowledge.

## Conclusions

NAMHS provides the first nationally representative data for mental health service use among adolescents aged 10–17 years in Kenya, Indonesia, and Vietnam. These findings highlight critical issues for governments, policymakers, and relevant stakeholders, i.e., that only a small proportion of adolescents with a diagnostic or subthreshold mental disorder accessed mental health services indicating a potentially substantial unmet need within the adolescent populations within these countries. This unmet need may result in the mental health problems experienced by adolescents persisting or increasing, interfering with their growth and development, productivity, and quality of life in the long term [39, 71]. As such, these findings provide critical impetus for governments and relevant in-country stakeholders to prioritize greater uptake and accessibility of adolescent mental health services provided through the heath sector while simultaneously harnessing the capacity of other sectors to increase access to high-quality mental health services for adolescents.

## Electronic supplementary material

Below is the link to the electronic supplementary material.

Supplementary Material 1.

## Data Availability

The NAMHS datasets are co-owned by the University of Queensland and each respective in-country lead organisation (K-NAMHS: APHRC and UQ; I-NAMHS: UGM and UQ; V-NAMHS: IOS and UQ). Currently, these datasets or analysis of these datasets are available for collaborative work on request to the relevant data owners following an established protocol. Work is currently underway to convert the NAMHS datasets into public use datasets, allowing for wide use of these datasets while ensuring protection of participant privacy, adherence to country-specific legislation, and appropriate use of data. This includes development of accompanying meta-data, inclusive of a codebook, technical manual, and analysis files. The expected launch date for these public use datasets and accompanying meta-data is 2026, with hosting mechanisms currently under development in line with country legislation and ethical requirements.

## Abbreviations

ADHD: attention-deficit/hyperactivity disorder
aOR: adjusted odds ratio
BHRC: Brazilian High-Risk Cohort
CI: confidence interval
DISC-5: Diagnostic Interview Schedule for Children, Version 5
DSM-5: Diagnostic and Statistical Manual of Mental Disorders, Fifth Edition
GAD: generalised anxiety disorder
GBD: Global Burden of Disease Study
HICs: high-income countries
HIV: human immunodeficiency virus
LMICs: low- and middle-income countries
MDD: major depressive disorder
NAMHS: National Adolescent Mental Health Surveys
NCS-A: National Comorbidity Survey-Adolescent Supplement
NHANES: National Health and Nutrition Examination Survey
NSDUH: National Survey of Drug Use and Health
PTSD: posttraumatic stress disorder
UHC: universal health coverage
