# Supplementary material for: Mental health service use among adolescents in three low- and middle-income countries: An analysis of the National Adolescent Mental Health Surveys
Source: Child Adolesc Psychiatry Ment Health. 2025 Jul 31;19(Suppl 1):84. doi: 10.1186/s13034-025-00924-2 (PMC12312253; doi:10.1186/s13034-025-00924-2)
Supplement: Supplementary file 1 — Supplementary Material 1. [file 13034_2025_924_MOESM1_ESM.docx]

Table S1. Unweighted numbers of adolescents who used any service providing support or counselling for emotional and behavioural problems in the past 12 months by mental disorder status in Kenya, Indonesia, and Vietnam.

| **Mental disorder status** | **Kenya** | | **Indonesia** | | **Vietnam** | |
| --- | --- | --- | --- | --- | --- | --- |
|  | **n** | **N** | **n** | **N** | **n** | **N** |
| Overall | 485 | 5,126 | 89 | 5,626 | 387 | 5,899 |
| Any mental disorder | 85 | 632 | 15 | 318 | 15 | 186 |
| Any subthreshold mental disorder | 208 | 1,686 | 31 | 1,722 | 81 | 997 |
| No mental disorder | 192 | 2,828 | 43 | 3,586 | 291 | 4,716 |
| Omitted* | 9 | | 38 | | 97 | |

*Omitted due to giving a non-meaningful response (i.e., Don’t know or Prefer not to say) to the question regarding use of any service providing support or counselling for emotional and behavioural problems in the past 12 months.

Table S2. Unweighted numbers for service use by adolescents with any mental disorder or subthreshold mental disorder in the past 12 months by key characteristics in Kenya, Indonesia, and Vietnam.

| **Characteristics** | **Kenya** | | **Indonesia** | | **Vietnam** | |
| --- | --- | --- | --- | --- | --- | --- |
|  | **n** | **N** | **n** | **N** | **n** | **N** |
| Total | 293 | 2,318 | 46 | 2,040 | 96 | 1,183 |
| Age   - 10-14 years - 15-17 years | 177  116 | 1,545  773 | 26  20 | 1,308  732 | 64  32 | 790  393 |
| Sex   - Male - Female | 103  190 | 1,116  1,202 | 24  22 | 987  1,062 | 56  40 | 592  591 |
| Urbanicity   - Rural - Urban | 170  123 | 1,075  1,243 | 31  15 | 1,320  720 | 31  65 | 501  682 |
| Wealth quintile   - 1 – lowest, least wealth - 2 - 3 - 4 - 5 – highest, most wealth | 20  38  57  97  81 | 271  391  494  579  583 | 16  7  5  8  10 | 484  415  466  358  317 | 18  15  31  7  25 | 199  237  315  168  264 |
| Mental disorder type ^a^   - Externalising only - Internalising only - Both | 101  101  91 | 639  1,075 604 | 5  28  13 | 325  1,328  387 | 10  77  9 | 97  974  112 |

^a^ In this instance, mental disorder includes both mental disorders and subthreshold mental disorders. Internalizing mental disorders include social phobia, GAD, MDD, and PTSD. Externalizing disorders include ADHD and conduct disorder. Adolescents could have more than one mental disorder.
